# Conscious Control over Action

**DOI:** 10.1111/mila.12082

**Published:** 2015-06-03

**Authors:** Joshua Shepherd

**Affiliations:** Faculty of Philosophy, Oxford Centre for Neuroethics, University of Oxford

## Abstract

The extensive involvement of nonconscious processes in human behaviour has led some to suggest that consciousness is much less important for the control of action than we might think. In this article I push against this trend, developing an understanding of conscious control that is sensitive to our best models of overt (that is, bodily) action control. Further, I assess the cogency of various *zombie challenges*—challenges that seek to demote the importance of conscious control for human agency. I argue that though nonconscious contributions to action control are evidently robust, these challenges are overblown.

## Introduction

Imagine losing conscious control over your own body, as happens (temporarily) when agents lose proprioception. Or imagine losing conscious control over your own thoughts, as seems to happen in certain cases of schizophrenia. The oddity and horror of the prospect indicates how deeply ingrained is our sense that the operations of mind and body are both, in some sense, subject to our own conscious control. Indeed, that agents routinely exercise conscious control is a truism of folk psychology, and one central to our self-understanding.

Recent cognitive science counsels caution—and according to some, scepticism—regarding this truism. Some, interpreting Milner and Goodale's (1995) important work on the visual system, have suggested that an important class of actions are controlled by nonconscious states and processes (Koch and Crick, 2001; Clark, 2007; Wu, 2013a). Suhler and Churchland (2009) have argued that a scientifically informed view of agency should make room for the work of non-conscious control. In their view, ‘Nonconscious control can be—and frequently is—exercised, and this control can be every bit as genuine as the conscious variety’ (p. 346).

It is undoubtedly true that nonconscious processes contribute much to routine exercises of control. But in my view claims about nonconscious control move too fast. Such claims are interesting in part because of a contrast with conscious control. But no general account of conscious control exists, and indeed, it seems to me that the phenomena, like many picked out by folk psychological concepts, is not well understood.

The nature of conscious control over overt action (that is, bodily as opposed to entirely mental action) is the main topic of this article. In Section 2 I elucidate a general model of overt action control, drawing on relevant work in the cognitive science of action. In Section 3 I consider the relation of consciousness to overt action control, and I develop a way of thinking about conscious control that is consistent with the relevant science, and sensitive to the elucidated model. In Section 4 I assess the cogency of various *zombie challenges*—challenges that seek to demote the importance of conscious control for human agency. I argue that though nonconscious contributions to overt action control are evidently robust, these challenges are overblown.

## Overt Action Control

Our knowledge of the workings of the human action-production system is far from complete.^1^ But at a certain level of abstraction, broad consensus exists regarding how an agent exercises overt action control. The general model begins with a computational picture of the way a system achieves behavioural goals in real time (Jeannerod, 1997; Wolpert and Kowato, 1998). Consider a specification of some goal, G. On the model, G is sent to an inverse model (or ‘controller’), which performs a computation over G and outputs a motor command M designed to drive the system towards the goal state. A copy of M is sent to a forward model (or ‘predictor’) which performs a computation over M and outputs a prediction P concerning the likely consequences of implementing M—that is, the likely feedback the system will receive if M is implemented. Throughout action production, the inverse model, the forward model, and the mechanism(s) responsible for maintaining and updating the goal-state receive updates from various comparator mechanisms. One hypothesised comparator mechanism compares the goal-state with feedback from the environment, and informs the inverse model of any errors; a second compares the goal-state with the forward model's predictions, and informs the inverse model of any errors; a third compares the forward model's prediction with feedback from the environment, and informs the forward model (so as to develop a more accurate forward model).

Call the mechanisms dedicated to achieving a goal a *comparator loop*. It is important to realise that for any given action numerous goals must be achieved by the action-production system, thus involving numerous comparator loops. Consider a simple action, such as reaching for a cup of coffee. Kathleen Akins' description of such an event is apt.

What directs your movements, from the moment you begin to lean forward in anticipation of reaching with your arm to the moment the coffee cup makes contact with your lips, is a host of information from numerous different sources. There will be visual information about the egocentric position of the cup relative to your body, about its position relative to your reaching hand, about the shape of the handle relative to your grasp, about the cup's rotation (shape) relative to a horizontal plane as you pick it up (do not spill it now), and about the cup's speed of movement; there will be proprioceptive information about the position of your upper body as you lean forward, about the angles of your arm joints as you stretch toward the cup, about the weight of the cup relative to the firmness of your grip, about the fantastically minute adjustments of your fingers, hand, and arm muscles as you balance the cup of liquid in an upright position, about the position of the cup and your hand relative to your lips (after all, you do not have to stare cross-eyed to get the cup there); there will be tactile information about the pressure of the cup in your hand, the pressure of the cup on your lips, the shape of the cup in your hand. No doubt this is but a small part of what is actually involved in the "simple" activity of picking up a cup of coffee (Akins, 1996, pp. 353–4).

Note the wide range of goals a system might need to achieve: goals concerning hand shape, grip strength, grip size, body posture, arm position, to say nothing of higher-level goals such as lifting the cup and sipping the coffee. We need not imagine that every possible goal is represented by the system as to-be-achieved: the system might use what shortcuts its design and its representational capacities allow. But it is easy to see that the production of many overt actions will require many such ‘comparator loops’ operating in a coordinated fashion, with the achievement of numerous functionally specific goals subserving the achievement of higher-level goals (or intentions). In line with general predictive coding models of the brain (see Clark, 2013), current sensorimotor control theorising posits a hierarchical architecture of comparator loops for overt action control (Grafton and Hamilton, 2007).

For a particularly good example of this, consider the control of speech. Saying almost anything—for example, ‘ugi’—requires the complicated coordination of higher and lower level goals involved with what the speaker intends to communicate, as well as how the speaker must coordinate breath, tongue, lips, and so on in order to make the required sounds. Consider the *motor* control of speech for a moment.^2^ On Joseph Perkell and colleagues' influential model (Perkell *et al*., 1997; Perkell *et al*., 2000), word-sounds are produced by way of goals that represent segmental components of sounds as spatio-temporal auditory regions. At this level, various comparator loops aim to produce various acoustic events, and the comparator loops become efficient by learning the relationships between various configurations of the mouth and vocal-tract in speech and the acoustic consequences that follow.^3^

Two important aspects of overt action control arise from the general picture painted thus far. First, on this general model feedback plays a crucial role. Feedback comes in two basic forms—sensory and predictive—depending on the mechanism providing it. Importantly, the action production system is somewhat flexible in its reliance on various sensory modalities. For various action-types, any form of sensory feedback could be relevant. We know, for example, that for many familiar action types both visual and proprioceptive feedback are important, and that their relative importance for a given action depends on specifics of that action. According to the optimal integration model developed by Robert van Beers and colleagues (van Beers *et al*., 1999; van Beers *et al*., 2002), the localisation of one's hand in action depends on both visual and proprioceptive cues. Importantly, the weighting of these two types of cues depends on contextual information. While it is often the case that vision dominates proprioception, proprioceptive cues receive more weight in conditions of self-generated movement, as well as conditions of degraded visual information. Of course, as we have already seen, there are action-types for which neither vision nor proprioception are crucial. Auditory feedback is most critical for the motor control of speech. For other action-types—e.g. sipping a hot drink—haptic feedback will be crucial. So it seems that for action control in general feedback is not only important, but the modality of the feedback depends upon the action-type in question.

This way of framing things emphasises the importance of learning for the utilisation of feedback. The thought is that over time the action-production system builds models of how an action goes, and these models will utilise the kinds of feedback that are typically important for the action-type. This is true as far as it goes. For example, agents who lose hearing late in life will continue to speak normally for some time, thanks to the robustness of their internal models—they retain the capacity for simulating and thus predicting auditory feedback even when they stop receiving such feedback. But it is important to note that the utilisation of feedback is plausibly structured by more than learning. Plausibly, particular intentions play a structuring role that guides the ways the system utilises feedback. Consider, for example, two different instances in which I produce a sentence. In one case I intend to articulate the sentence clearly. In another I intend to shock you by uttering the sentence (which, it turns out, is an off-colour remark). Though the acoustic goals in the two cases will be very similar, in the latter case visual feedback of your face will likely receive greater weight, while in the former auditory feedback (and probably proprioceptive feedback from the vocal-tract and tongue) will receive greater weight. At least at some levels of the comparator-loop hierarchy, the top-down structuring influence of intentions likely plays an important role in what feedback is deemed relevant,^4^ and thus in how the agent exercises control in a given instance.

Evidence for this last claim can be found in recent work on the ways that action influences perception. For example, Maruya *et al*. (2007), put participants in a visual condition of binocular rivalry. In this condition, one eye saw a sphere full of dots that, thanks to the motion of the dots, appeared to rotate. The other eye saw a sphere of black and white grating that alternated and thus gave the sphere a flickering appearance. In normal conditions of binocular rivalry, conscious vision will oscillate between stimuli. But in this experiment Maruya *et al*. gave participants a way to control the motion of one sphere: by dragging a mouse across the sphere full of dots participants would control the rotation of that sphere. The result: ‘for each observer, manual control of the motion of the sphere lengthened dominance durations of the sphere, relative to the durations on automatic trials, and manual control abbreviated suppression durations of the sphere, again relative to the durations on automatic trials’ (p. 1094). Maruya *et al*. conclude: ‘conflict between two incompatible visual stimuli tends to be resolved in favour of a stimulus that is under motor control of the observer viewing that stimulus’ (p. 1095). In this case, the agent's intentions have a structuring influence on the utilisation of feedback.^5^

Second, notice that as typically elucidated, models of overt action control explain how the agent moves the body to achieve a given goal in real time. In so doing, these models often take a relatively well-specified goal-state as a given. But in fact the intentions and goals sustaining and guiding human activity are often—though not necessarily always—*open-ended* in an important way. The environment is messy: in order to satisfy an intention, agents often need to *fill it in* as an action proceeds. And the environment is unfriendly: in order to achieve their goals, agents often need to *revise* their intentions as an action proceeds. Implementation is not all there is to overt action control: there is an important place for fill-in and revision as well. I will call the processes of planning, plan filling-in, plan revision, and plan monitoring executive aspects of overt action control. I will call the processes that transform a relatively well-specified goal state into states of affairs that satisfy the goal state implementational aspects of overt action control.

To get a feel for the implementational/executive distinction, consider Peter engaged in an extended bout of activity—a swordfight with Hook. Suppose that Peter intends to defeat Hook, and intends to do so by way of a relatively open ended plan (e.g. defeat Hook by baiting him into opening up his left side, then capitalising). In order to carry out this plan, Peter must implement it by way of a number of fine-grained movement sequences. Lunge, sidestep, thrust, adjust grip on the sword, duck, slide—these are all implementational aspects of overt action control. In addition to executing such elements, Peter must monitor the progress of his plan. Likely, Peter will need to fill in, revise and adapt the plan guiding his action in certain ways, in response to the evolving swordfight. It is possible that injuries to himself or to Hook, the introduction of new elements in the nearby environment, and so on, will come to impinge on the feasibility of his general plan. At some point in the swordfight, Peter may need to revise his strategy for defeating Hook, so he may need to revise his guiding intention. Peter may also need to totally change his intention, from an intention to defeat Hook to, perhaps, an intention to escape death, or an intention to protect an innocent bystander. Peter's monitoring and adjusting his plan are executive aspects of overt action control.

Given the unfriendliness and messiness of most action contexts, the interaction of well-functioning comparator loops—at whatever level of the action control hierarchy—will require both implementational and executive aspects. But it is plausible to posit relative differences in the importance of these aspects, depending on the place of a given process in the action control hierarchy. In general, the most critical executive processes occur towards the top of the action control hierarchy, where it is more appropriate to speak in terms of interactions between desires, beliefs and intentions. And the most critical implementational processes occur towards the bottom of the action control hierarchy, where it is more appropriate to speak in terms of interactions between motor commands, predictive signals, and error signals. So in general, executive aspects will be more important at higher levels, and implementational aspects will be more important at lower levels. As a rule of thumb, we might say that at higher levels it is most important to get the plan right, while at lower levels it is most important to get the plan done. Since implementational and executive aspects of overt action control occur at all places in the hierarchy, the distinction I am drawing is not sharp. Nonetheless, in what follows it will be useful to speak of implementational and executive *dimensions* of overt action control, where the implementational dimension is understood as occupying (roughly) the lower end of the hierarchy, and where the executive dimension is understood as occupying (roughly) the higher end of the hierarchy.^6^

Although many details concerning the relation between the executive and the implementational aspects of overt action control remain unknown, there is evidence that the distinction tracks functionally important features of the action-production system. Consider, for example, the well-studied differences between ideational and ideomotor apraxia. Patients with ideomotor apraxia have difficulty in basic motor performance (Roy and Square, 1985)—they suffer primarily from impairments in action implementation. By contrast, patients with ideational apraxia have difficulty with the more conceptual aspects of action, e.g. in understanding tool-action relationships (Ochipa *et al*., 1992). They suffer from impairments in action planning.

Control-related differences between schizophrenic and autistic agents likewise appear to track the implementation/executive distinction. Although schizophrenic patients are unimpaired in their ability to perform low-level motor control tasks, such as maintaining a consistent matching relationship between forces exerted with either hand (Lafargue *et al*., 2006), or tracing a line (Fourneret *et al*., 2001), such agents suffer impairments in higher-level aspects of action control such as the production of motor imagery (Danckert *et al*., 2002) and the translation of conceptually laden action-goals into motor performance (Lafargue *et al*., 2006). Schizophrenics seem to suffer a selective impairment of executive aspects of overt action control.

Although the issue is controversial (see fn. 7), autistic agents might suffer the opposite impairment. Although action planning is largely intact in this population, a recent study shows autistic agents have difficulty implementing action-plans that require the chaining of multiple motor schemata (Stoit *et al*., 2013).^7^

There is evidence, then, for a *functional dissociation* between executive and implementational aspects of overt action control. But there is also evidence for *functional integration*. We have already seen some—the Maruya *et al*. (2007) study. There, executive involvement in action, via an agent's intention to control the motion of a sphere, played a subtle role in structuring the way low-level sensory feedback was utilised for control of what the agent was doing.

Further evidence for this general point comes from work on sensory attenuation. Sensory attenuation occurs when, roughly, there is a lower degree of felt or cortically measured intensity for some sensory stimulus. Functional magnetic resonance imaging confirms a difference in primary somatosensory cortex for self-generated versus externally generated tactile stimuli (Helmchen *et al*., 2006). Work using electro encephalogram (EEG) demonstrates reduced amplitudes in auditory event-readiness potentials (ERPs) for self-generated versus externally generated auditory stimuli (Martikainen *et al*., 2005). In general, it is thought that sensory attenuation is related to motor control: in self-generation conditions, the motor control system is better able to predict upcoming stimuli, and to shift attention away from these stimuli for use elsewhere. It might be thought, then, that sensory attenuation has only to do with implementational aspects. But a recent EEG study by Gentsch and Schütz-Bosbach (2011) found that the N1 component of visual ERPs was attenuated both in conditions of self-generation and of cognitive (i.e. prime-induced) expectation of the visual stimuli, as compared to conditions of passively viewing the stimuli. Since an agent's expectations have little direct effect on the motor implementation of an action goal, this would seem to indicate that executive aspects are involved in the implementational process of sensory attenuation. Further evidence for this view comes from Desantis *et al*. (2012), who demonstrated that beliefs about whether a tone was self-produced or not also led to sensory attenuation. Finally, a recent study by Timm *et al*. (2013) showed that voluntary button presses which produced an expected auditory effect led to attenuated auditory ERPs, while transcranial magnetic stimulation of the motor cortex, which also produced a button press and an auditory effect, did not lead to attenuation. The only relevant difference between these cases is, of course, the involvement of executive aspects in the voluntary condition. Again, this is evidence that executive involvement in action plays subtle and important roles in structuring the uptake of sensory feedback during action control.^8^

To summarise, overt action control appears to be subserved by (a) largely executive dimension processes that utilise cognitive and conceptual resources to construct, fill in and revise the content of the intentions that guide behaviour, and by (b) largely implementational dimension processes that are relatively dependent on the specification of various goals, in the sense that these processes aim to achieve given goals in real time. Although these dimensions are functionally dissociable, they are also highly integrated, such that on-line overt action control appears to require the efficient operation of executive and implementational processes at higher and lower levels, and *in tandem*. In a sense this is predictable. An agent's planning must be sensitive to her implementational context—to what the environment affords as well as to what she is able and knows how to do. I have reviewed evidence that suggests that an agent's implementation is also sensitive to her planning. In particular, there is evidence that an agent's executive dimension states and processes—i.e. the higher-level intentions, expectations and goals guiding a particular action—play a role in structuring lower-level utilisation of sensory feedback.

This general picture of how human agents exercise overt action control constrains what we can plausibly assert about the conscious control human agents purportedly exercise. In the next section I develop a way of thinking about conscious control that is sensitive to the relevant constraints.

## A Model for Conscious Control

We are interested in the conscious control human agents might exercise over action. Regarding humans, all exercises of overt action control require some contribution from non-conscious states and processes. And although I examine some counter-proposals in Section 4, it is difficult to find a genuine example of remotely sophisticated overt action control executed with no contribution from conscious states and processes. Most human exercises of overt action control require rich collaboration between conscious and non-conscious states and processes.

Suppose for the moment this is right. What are the implications for our understanding of conscious control? One might find it natural to conceptualise conscious control as a type of overt action control for which conscious states and processes are *in charge*. This has an intuitive ring, but is problematic. When exercising control the agent is in charge of her behaviour. But it is too strong to identify the agent with the conscious states and processes that contribute to exercises of control.

Alternatively, one might argue that conscious control is primarily a matter of executive elements—perhaps of some executive ‘system’—driving the operations of an implementational ‘system.’ This seems to cohere with a commonsense way of conceptualising the role of the (metaphorical) conscious self in action. On this conceptualisation, the conscious self is identified primarily with executive processes—indeed, commonsense often seems to identify the conscious self with a subset of executive processes, i.e. with processes of practical reasoning—and these processes are collectively thought of as the captain of the ship, the CEO of the corporation, or whatever. Again, this way of thinking is intuitive, and coheres with our experience of agency in at least some circumstances. But this way of thinking about conscious control suffers from at least two problems. First, it tends to assume that conscious processes are primarily responsible for the operations of an executive system—practical reasoning is thought to be a primarily conscious endeavour. This neglects data that suggest that executive processes depend on nonconscious processes as well (e.g. Cresswell *et al*., 2013), and thus obscures the fact that we need an account of what consciousness does *for* the relevant executive aspects. Second, this way of thinking neglects the possibility that conscious processes are important for implementation as well, and that the conscious implementation of an action plan is an important type of conscious control in its own right.

In my view, it is better to think of conscious control over overt action as a subset of the broader class of overt action control. An exercise of conscious control, on this view, is an exercise of overt action control for which conscious states or processes make critical causal contributions. Importantly, on this understanding, conscious control is two-dimensional in the following sense: for a given action-type, one might exercise conscious control along the executive dimension or along the implementational dimension (or along both). Whether a given action-type *requires* conscious contributions along either the executive or the implementational dimension is not an issue I can address here. Plausibly, a wide range of local factors will influence the need for or uptake of conscious processes within the broader action-control system: the importance of consciousness along either the executive or implementational dimension might vary depending on the action-type, features of the action as it progresses (i.e. the part of the action-plan currently being implemented), on circumstances internal to the agent (such as energy levels, attentional saturation, skill level regarding the action-type), on circumstances external to the agent (whether the environment is resource-rich regarding the successful performance of the action or not), and so on. So it will not necessarily be true that an agent exercises a higher degree of control over an action A only if she exercises more (implementational and/or executive) conscious control. One basic finding from the literatures on skill and automaticity is that as expertise in some domain increases, the need to focus conscious attention on certain segments of actions in that domain decreases. This does not entail that consciousness becomes unnecessary or unimportant. The point is simply that *various aspects* of consciousness are seemingly often needed *to various degrees* along *either* the implementational *or* executive dimensions.^9^

I have deployed the notion of a *critical* causal contribution in demarcating conscious control from overt action control more generally. This is because I find it insufficient to require merely that consciousness make some causal contribution—some causal contributions are insignificant. But how should we understand what makes a causal contribution critical? I prefer to understand this notion in terms of degrees of control. When we ask whether consciousness was causally important for some exercise of control, often we want to know whether some exercise of overt action control was performed more *smoothly* or more *effectively*, or whether the exercise was in some sense *better* because of some causal variable of interest.^10^ This requires specifying a relevant contrast class, and requires in addition a general account of the degrees of control, and what enhances or diminishes them. In other work I have offered such an account (Shepherd, 2014). There is not space to review it here: it will suffice to characterise key features of that account, and apply them to the case of conscious control.

In my view, an agent exercises a higher degree of control over an instance of behaviour B to the extent that B more closely matches the representational content of the mental state(s) guiding B, and so long as the causal pathways producing B involve no deviant causation (I offer an analysis of non-deviant causation in Shepherd, 2014). Paradigmatically, the relevant mental state will be an intention, but in many cases desires, beliefs, and related states will be relevant as well. In order to determine whether some causal variable—in this case, some conscious state or process—makes an important difference to an exercise of control, then, we consider a set of relevantly similar circumstances across which the variable contributes, and we consider a contrasting set of relevantly similar circumstances across which the variable is absent. At minimum, we do this by holding fixed the content of the relevant mental states, as well as by specifying the kind of circumstances in which the agent is behaving (for discussion regarding various useful ways of doing this, see Shepherd, 2014, §3). This gives us a way to understand the importance of some variable's contribution. Roughly, a variable makes a critical contribution to the extent that the agent exercises a higher degree of control over her behaviour across the set of circumstances that includes the variable.

Granted, we are not often in position (yet) to measure the impact of some conscious state or process on a temporally extended process of overt action control. Even so, this account gives us a way to think about conscious control that is consistent with current knowledge of overt action control, and with the very plausible view that overt action control depends on contributions from conscious and nonconscious processes working in tandem. And there are less precise ways of explicating this language of degrees of control that might nonetheless prove useful. For example, it is consistent with what I have said above to maintain that some aspect of consciousness (e.g. some conscious state or process) makes a (sufficiently) critical contribution to control over some action if in the absence of that aspect the agent's performance noticeably suffers.

I now focus on recent challenges to the claim that consciousness sometimes makes critical causal contributions to exercises of overt action control. I wish to argue that these challenges, as presently constituted, fail. We have no good reason to think that consciousness does not make critical causal contributions to overt action control, and we have some reason to think that in some cases, consciousness does make critical contributions (along both executive and implementational dimensions). Or so I will argue.

## Zombie Challenges

In this section I outline two challenges to the intuitive view that consciousness is causally important for overt action control. Call these zombie challenges: arguments that purport to show that though it seems to us that overt action is controlled by conscious states and processes, in fact overt action is controlled by nonconscious (zombie) states and processes. Attention to a few aspects of a zombie challenge is necessary. First, the challenges I discuss below are all *partial* challenges in the sense that they do not endorse philosophical epiphenomenalism. Consciousness is assumed to make some causal impact on behaviour—the general claim is that the causal impact is surprisingly insignificant. This is a claim about the degree of consciousness's involvement, and given the current state of knowledge, replies will be about the degree of consciousness's involvement.

Second, it is important to be clear about the structure of a zombie challenge. On my interpretation, two claims are essential. The first claim appears, at first glance, to be phenomenological: it seems to us that overt action is controlled by conscious states and processes. However, here I want to resist an exclusively phenomenological reading of this claim. It will be enough if a zombie challenge runs counter either to our phenomenology or to our *beliefs* about phenomenology. Zombie challenges are supposed, first and foremost, to be *surprising*. An account that undermines phenomenology or widely held beliefs about it will suffice.^11^ The second claim is broadly causal: overt action is controlled by nonconscious states and processes (or rather: overt action depends almost entirely on critical causal contributions from nonconscious processes). Often the second claim receives the most attention—sometimes, the first is no part of the zombie view put forward. But without the first claim the second is much less interesting. Consider two cases of overt action control, 1 and 2. In 1, paying close attention to what you are doing, you intend to move your arm in a certain way and then slowly do so. If you are like me it seems that conscious states and processes make important causal contributions to both the content of your plan and the implementation of your plan. In 2, you begin moving your arm more quickly and freely, without paying much attention to what you are doing. If you are like me, after a short while it seems as though conscious states and processes make very little if any contribution to the movements you make. You continue moving your arm, but it becomes possible to do something else entirely, focusing very little if at all on what your arm is doing.

It would not be surprising to discover that consciousness played little to no role in controlling the movements of your arm in case 2—there is no phenomenology of conscious control in that case, nor do we have any beliefs to the effect that consciousness is important in such cases. What would be surprising is if consciousness played little to no role in case 1. The moral generalises. We want to know whether consciousness makes critical causal contributions to a certain class of exercises of overt action control, namely, the class for which it seems (either phenomenally or epistemically) that consciousness makes critical causal contributions. Now, in my view the contours of this class are not entirely clear—we need a better phenomenology of agency. But arguably, we can make a beginning by examining cases in which consciousness seems *clearly* to make some contribution, and by asking what aspect of consciousness is at issue and what that aspect's causal contribution might plausibly be.

If one of the zombie challenges I examine below succeed, the results of this process will look rather grim. According to a zombie challenge, either our beliefs or our phenomenology are mistaken. In what follows I examine two zombie challenges: one from vision-in-action, and one from expertise.

### Vision-in-Action

Perhaps the most strident zombie challenge stems from work on the human visual system. This work suggests an important functional distinction in the human visual system, between the dorsal stream and the ventral stream.^12^ According to Milner and Goodale's influential dual visual systems hypothesis, these streams handle fundamentally different kinds of information. The dorsal stream handles egocentric visual information, is responsible for on-line overt action control, and is not involved with conscious vision. The ventral stream handles allocentric visual information, is not responsible for on-line overt action control, and is largely responsible for conscious vision.

Some evidence for this distinction stems from patients with lesions to either the dorsal or the ventral stream. Patients with lesions to the ventral stream display visual agnosia. Although they cannot consciously recognise features of objects such as their shape or spatial orientation, these patients retain the ability to control overt actions related to objects. The patient DF, for example, will fail to consciously recognise the orientation of a slot. But when asked she will post a letter through the slot, rotating her wrist in just the right way as she does so. Conversely, patients with lesions to the dorsal stream display optic ataxia. Though a patient with optic ataxia will consciously recognise a cup on a table, attempts to grasp the cup will display seriously diminished control.

Further evidence for the dual visual systems view stems from work on healthy human adults. One basic finding is that illusions regarding the size of consciously perceived objects often have little influence on actions related to the objects. When presented with two identically sized circles, each surrounded by a ring of differently sized circles, participants reliably judge one circle larger than the other. Yet, when asked to grasp the circles, participants' grip apertures reliably match the actual size of the circles (Agliotti *et al*., 1995). So it looks like the ventral stream processes responsible for the conscious visual illusion are out of the action-production loop, while the dorsal stream processes are both responsible for the fine-tuned modulation of grip size and unresponsive to the conscious visual illusion.

Though proper interpretation of this work remains controversial (see, e.g., Schenk and McIntosh, 2010), reflection on this work has led to the endorsement of zombie theses of various kinds. Milner and Goodale themselves have claimed that ‘the visual system that gives us our visual experience is not the same [visual] system that guides our movements in the world’ (2004, p. 3). Many philosophers have agreed. Andy Clark (2007) has denied Morgan Wallhagen's (2007) claim that ‘conscious visual experiences are typically utilized in the control and guidance of voluntary/intentional behaviours’ (Clark, 2007, p. 573). Berit Brogaard (2011) has argued that the visual states that control action—she calls these ‘action-guiding dorsal stream representations’ (Brogaard, 2011, p. 1078)—are not cognitively accessible, and do not correlate with phenomenal consciousness. Wayne Wu (2013a) has argued for the thesis that ‘some visual representations that directly control and guide mundane bodily actions are unconscious’ (2013a, p. 218).

A detailed treatment of the conscious vision-in-action literature would take another paper (or more). I limit my response here to a few (I think crucial) observations. First, these zombie theses have thus far given insufficient attention to the phenomenology of overt action control. The kinds of movements for which dorsal stream processing is critical are very fine-grained. They include adjustments of grip aperture at the level of millimetres. Is there any phenomenology of vision informing such fine-grained adjustment? It is dubious there is. In his (2007) Andy Clark acknowledges something like this point, but argues that the non-involvement of conscious vision is radical enough to undercut what phenomenology there might be. In short, though conscious vision *appears* to present the world in a way ‘especially apt for, and typically utilized in, the control and guidance of fine-tuned, real-world activity’ (Clark, 2001, p. 496), it turns out that not just fine-tuned adjustments but ‘even the gross heading and kinematics’ are programmed ‘by the distinct representational structures proper to the dorsal stream’ (Clark, 2007, p. 573).

As Clark makes clear in that paper, this is a claim about degrees of consciousness's involvement. There is reason to believe that the ventral stream plays roles in supporting the control of some overt action-types—for example, action-types that require object-recognition (MacIntosh and Lashley, 2008), and action-types that are unpracticed by the agent in question (Gonzalez *et al*., 2008). More recent evidence indicates a role for ventral stream programming even in the rapid response to environmental change during an instance of overt action control. Caljouw *et al*. (2011) had participants hit a ball towards the vertex of a Müller-Lyer illusion, across various conditions: a condition in which the illusion remained stable throughout the activity, and conditions in which the illusion was perturbed (by flipping the tails of the Müller-Lyer arrows) at some point after the hitting motion had already begun. They found that the perturbations significantly influenced control of the hitting actions: mean impact velocity of the hitting device with the ball changed in the direction of the (consciously) perceived illusory change of the target. As Caljouw *et al*. report, ‘any abrupt environmental change in the target area invoked online visual control that is affected by the illusion. In other words, it seems that egocentric information is used to visually guide limb movements to interact with a stable non-changing target, whereas allocentric information is used to adjust ongoing limb movements in response to sudden target perturbations’ (p. 1139). This study casts doubt on any degreed statement about conscious vision's involvement. For the execution of overt action control will often require adequate response to environmental perturbation.

A further possible role for conscious vision in overt action control tracks the distinction between executive and implementational dimensions of overt action control. Clark sets up his zombie challenge along a similar distinction: he argues that while conscious vision is important for the (executive) activity of action-selection, it is unimportant for the (implementational) activity of action-control. But as I have argued, executive processes do not cease functioning at the moment of action-initiation—given the incompleteness of many intentions, and the unfriendliness of many environments, executive resources are needed throughout the entire process of overt action control.

In this connection, consider an interesting study on putting. To interpret this study, it is important to know that recent research on motor performance in aiming tasks has uncovered the importance of ‘Quiet Eye Duration’, a term of art that indicates the duration of an agent's final fixation towards a relevant target before action-initiation. It turns out that experts exhibit longer Quiet Eye Duration than novices, and that no matter one's level of expertise longer Quiet Eye Duration is associated with higher rates of action-success in tasks such as putting, shotgun shooting, basketball shooting, dart throwing, and penalty kicks in soccer (see Mann *et al*., 2007 for a review). Further, Quiet Eye Duration is closely associated with visual attention. Since visual saccades must be planned, it is hypothesised that the termination of the Quiet Eye (the visual fixation) is preceded by a shift in covert attention to the site of the planned saccade. As such, Quiet Eye Duration is often taken as an operationalisation of visual attentional control.

There is some debate concerning the role of Quiet Eye Duration, and some have claimed that its importance is primarily for the planning/motor programming of action, rather than for on-line control. To test this, Vine *et al*. (2013) had experienced golfers—the average handicap of the group was 3.6—attempt a series of 5-foot putts until they missed one. They measured the duration of Quiet Eye during putt planning (measured up until action-initiation), the duration during the actual putting motion, and the duration after ball strike, for three putts: the first made putt, the last made putt (i.e. the putt just before the miss), and the miss. The golfers made an average of 23 putts before the miss. Vine *et al*. found no significant effect for Quiet Eye Duration during putt planning across all three putts. But Quiet Eye Duration during the actual putt and just after the putt was significantly longer during the made putts. For the missed putt, duration during the putt decreased from more than 800 ms to 560 ms and duration after the putt decreased from 400 ms to less than 100 ms (Vine *et al*. 2013, p. 1992). As Vine *et al*. note, this supports a view on which visual information is important for the on-line control of movement. Further, given the close association of Quiet Eye Duration and visual attentional control, it looks like the failures reflected by lower Quiet Eye Duration on missed putts were failures of visual attentional control.

What does this have to do with consciousness? If Milner and Goodale are right, the on-line visual information that is important here is run primarily through the dorsal stream. I have challenged this thought, but the point here is different. It looks like visual attention is critical for overt action control (at least for putting). And while it is possible that nonconscious states are primarily responsible for visual attentional control, this result is not guaranteed. It is likewise possible that the control of visual attention is subserved by conscious processes. The suggestion is promising since here there is some relevant phenomenology. Anyone who has putted before will be familiar with the phenomenology of intentionally focusing (or of intentionally trying to focus) on the ball. Unless this phenomenology is mistaken—and there is thus far little reason to think that it is—it could be that intentional conscious visual focusing is critical for successful putting (and for successful action directed towards targets more generally). If so, this would be an example of a conscious process critically influencing the on-line control of overt action.^13^

### Expertise

I must confess that the challenge from expertise does not map perfectly onto my setup of the relevant issues. In large part this is because much is at issue in the science of expertise—there is more of interest here than the question of the causal involvement of consciousness in overt action control. But a kind of zombie challenge can be constructed from discussions of expertise. That this is possible I take to be of interest independently of how I respond to the challenge. That is to say, I suspect some enterprising theorists will find reason to disagree with my response and to ratchet up the constructed challenge.

The basic finding of note here is this. Though consciousness seems important for unskilled activities, as expertise increases it seems the need for consciousness falls away. Indeed, it is often noted that many studies suggest that at a certain point in development, consciously attending to details of implementation hinders performance (Beilock and Gray, 2012). Regarding these points, however, care is required. As Hubert Dreyfus interprets the data—and the phenomenology of skilled action—it is not the case that consciousness falls away entirely. Rather, a certain aspect of consciousness falls away. Consider Dreyfus's discussion of a Grandmaster playing lightning chess. ‘A chess Grandmaster facing a position … experiences a compelling sense of the issue and the best move … When the Grandmaster is playing lightning chess, as far as he can tell, he is simply responding to patterns on the board. At this speed he must depend entirely on perception and not at all on analysis and comparison of alternatives’ (2005, p. 53). As Dreyfus has it, consciousness is still important, or at least involved. The Grandmaster relies on a kind of fluid intuitive sense of what to do. What falls away are certain kinds of conscious executive processes—the kind that require concept-laden practical reasoning and explicit forms of attentional monitoring (compare also Railton's view of expert action in Railton, 2009). On this way of understanding the challenge, it exclusively applies to conscious control along the executive dimension. We might say that the challenge from expertise consists in the claim that *expert control* of overt action requires no conscious influence along the executive dimension of overt action control, and indeed is often hindered by such influence.

In what sense is the challenge from expertise a *zombie* challenge? As typically articulated, this challenge relies on an absence of phenomenology. That is, the challenge is often articulated by way of quotes and reports from professional athletes to the effect that expert performance is unthinking performance. Consider Larry Bird's admission: ‘A lot of times, I've passed the basketball and not realized I've passed it until a moment or so later’ (quoted in Brownstein, 2013, p. 11). But if the absence of relevant phenomenology is used to set up the challenge, what about it is supposed to be surprising? Do we need scientists and philosophers to tell us about a purportedly clear feature of human agency?

Perhaps the surprising element comes from poorly formed beliefs about the role of consciousness in expert action. Perhaps we think that all paradigm intentional action depends upon consciousness in some way, and since expert action is paradigmatic, we expect that consciousness will be important in this domain as well. Perhaps: but I suspect that there is a hidden phenomenological element to this challenge as well. This is because, as I argue below, in fact even executive-dimension conscious processes are important for expert action. Since there is a phenomenology to expert action control, not only the data but also the reports of experts surprise us. Is it really true that so-called executive conscious processes fall away in expert action, and that a reintroduction of them hinders expert action?

There is reason to think that, even if expert control of overt action requires less from so-called executive conscious processes, it is not true that the importance of these processes is reduced to nil, nor that a reintroduction of these processes is necessarily a hindrance. Recall, initially, the Vine *et al*. putting study. In that study a drop-off in performance was closely associated with a loss of visual attentional control. Visual attentional control is best seen as executive in nature—attentional control is responsive to the agent's intentions and efforts, and biases sensory processing in a characteristically top-down manner (see Hopfinger *et al*., 2000). So we have already seen evidence indicating that executive (and plausibly, conscious) processes are important for at least some expert action.

A study by Guillot *et al*. (2013) offers further evidence. This study examined the impact of the use of motor imagery for high-jumping amongst elite high-jumpers (i.e. the high-jumpers had been competing in national-level competitions for over 5 years). Participants self-reported using motor imagery techniques, including those involving kinaesthetic and visual imagery (p. 3), suggesting that motor imagery was already of some benefit for their control over high-jumping actions. Guillot *et al*. studied the effectiveness of the explicit deployment of motor imagery in two conditions: one involved motionless motor imagery before the jump, and a second involved dynamic motor imagery (involving actual overt movements alongside imagery) before the jump. In the dynamic motor imagery condition the jumpers exhibited better performance along several metrics, including bar-clearance rate, and technique as measured by independent judges. Thus, conscious executive processing—here embodied by the use of motor imagery techniques—appears to improve implementation of an expert action. It is important to note that a proponent of the challenge from expertise would predict just the opposite of this. According to the challenge from expertise, expert action is supposed to be automatized to the degree that bottom-up processes need no (or very little) input from top-down, executive dimension processes. This study finds the opposite. Guillot *et al*. speculate that the difficulty of high-jumping might render it resistant to such automatisation: ‘moving while imagining is likely to enhance the mental representation and the calibration of the run-up, which usually remains a difficult task even in confirmed athletes. Indeed, athletes must resolve a complex relationship between the speed of the approach running and the vertical velocity to be produced for jumping. Moving while imagining might therefore contribute to stabilize a given tempo for this part of the whole movement. Practically, this result suggests dynamic imagery might be used regardless of the level of expertise’ (p. 6).

The use of motor imagery in this study occurred before the action in question. Doubtlessly, then, some will complain that I am ignoring an important class of actions—a class which neither the high-jumping study nor the putting study addresses. These would be actions that require rapid responding to features of the situation. Arguably, these are the types of actions motivating the challenge from expertise in the first place. So the complaint: why think executive-dimension consciousness is important for these types of actions?

In a recent paper David Papineau sharpens the worry. His focus is cricket, which has been fairly well studied in recent years. Two claims Papineau makes are salient here. First, consciousness (at least executive-dimension consciousness) is unimportant for the real-time control of batting in cricket: ‘there is no room for real-time conscious decisions in batting. Batting is automatic, not under conscious control. There is no time to think once the ball has been released. You can only react’ (Papineau, 2013, p. 177). Second, that consciousness is unimportant for real-time cricket batting in some sense contradicts the phenomenology of cricket batsmen, including experts. Regarding the finding that the eyes of cricket batters do not maintain contact with the ball throughout its flight, but saccade to the place they (subconsciously) predict the ball will hit the ground, Papineau comments:

To anybody who has played cricket, this will seem surprising, not to say incredible. The first thing that young batsmen are taught is to keep their eye on the ball. And certainly when you are actually batting, your awareness is of the ball moving continuously through the air from the bowler's release until it reaches you. When a distinguished Australian opening batsmen heard about the eye saccades at a conference, he started his contribution to the discussion with—‘I don't believe a word of it’ (2013, pp. 177– 8).

It must be stated here that the phenomenological reports of experts are often difficult to interpret. (Often, in my view, they are over-interpreted.^14^) Explicit practical reasoning culminating in a decision is not the only way executive processes might contribute to an exercise of overt action control. With many familiar actions, we come to possess a flexible know-how that permits influence on the action in subtle ways.^15^ Even so, the sport psychology of cricket batting must be taken seriously. The real-time control of rapid actions is too quick to leave much room for conscious influence. But then these actions are too quick to leave much room for nonconscious influence as well. Why think nonconscious processes are much more important than conscious processes for the real-time control of rapid actions?

One general reason often offered is that the nonconscious processes that support overt action control are in some sense faster than the conscious processes. It takes time for a stimulus to ‘reach consciousness’—too much time in the case of rapid actions. Another reason sometimes given appeals to the dual visual systems hypothesis considered in Section 4.1. Rapid actions depend more closely on the kind of fine-grained (and more rapidly responsive) action control supported by the dorsal, rather than the ventral stream.

I do not wish to counter either of these general thoughts. The key question is whether an important class of rapid actions exist which is too rapid for consciousness (at least, executive-dimension consciousness) to make a real-time critical causal contribution. Cricket seems a good test case: at the professional level there is roughly a half-second in between the release of the ball and its arrival at or near the bat. (Expert batters have a little more time than this. It is well established that expert batters use information from the bowler's wind-up to predict the direction of the bowl (Abernethy and Russell, 1987). So by the release point, the expert batter's attention will already be informed by generally accurate expectations.^16^)

Does the expert control of batting involve any contribution from executive conscious processes? Even if rapid changes are subserved primarily by dorsal stream or otherwise nonconscious processes, it is possible that conscious attentional control is involved. The fact that putting is a relatively slow action does not undermine the finding that attentional control is important for expert action. Batters are clearly—and it seems consciously—paying close attention to the incoming ball. Further, we have seen evidence that in conditions of a changing environment ventral stream processes might become important (Caljouw *et al*., 2011). There is not enough evidence to conclude anything with high confidence. But this applies to sceptical claims about conscious control in rapid action as well. It has not been shown that conscious executive processes play no significant role for expert rapid overt action control.

There is, however, a lingering worry about phenomenology. Is it true that expert batters have a systematically mistaken phenomenology? If it is, then one might question whether conscious attention could be of any use here. And one might take the evidence to indicate that phenomenology is mistaken. As Mann *et al*. (2013) note, there is something of a consensus in the field that ‘the ball moves too quickly for the eyes to be able to track it’ (p. 1). This is bad news for batters who assert a phenomenology of seeing the ball. However, Mann *et al*. (2013) offer some reason to be sceptical of this consensus (in fairness to Papineau, this study is novel and appeared after his accurate summary of the relevant literature).

They explored the ability of novice and high-level expert batters to track incoming balls. After discussing the phenomenology of batting with experts, Mann *et al*. report the following:

Justin Langer, a recently retired international batter (and more recently, the Australian Batting Coach) found the concept of not watching the ball hit the bat as unbelievable, as he clearly describes seeing markings on the ball as it makes contact with the bat (personal communication, 6/03/11). Further, a current international player, one of the top five international run-scorers of all time, reports that one of his key aims when batting is to watch the ball come out from underneath my bat when he hits it (personal communication, 9/18/11). It is highly unlikely that either of these tasks would be possible unless the ball was fixated using central vision at the moment of bat-ball contact (Mann *et al*., 2013, p. 2).

Mann *et al*. thus sought to explore whether this bit of the phenomenology—that of seeing the ball hit the bat—was supported by physiological data drawn from experts. The experts in their study were two Australian batters, each of whom had played in over 70 test matches for the Australian national team, averaging over 45 runs per inning (these are world-class cricket batters). And the results are telling. In contrast with novices, the expert batters in fact did keep their gaze aligned with the ball: ‘the elite batters directed their gaze ahead of the flight-path of the ball immediately prior to bat-ball contact, whereas the gaze of the club-level batters tended to be behind the ball. The elite batters appeared to use a strategy that ensured they could ‘park’ their gaze ahead of the ball so that gaze could ‘lie-in-wait’ for the ball to arrive’ (p. 6). The experts did so by, first, exhibiting superior skill at coupling the movement of their head with the motion of the ball, and second, by making more accurate predictive saccades both to the location where the ball would hit the ground, and then to the location of contact with the bat.

Of course, this study does *not* show that conscious processes are important for expert control over batting. But it does show that scepticism about the phenomenology of experts is unwarranted, and it uncovers some tantalizing prospects for future research. Here is how Mann *et al*. conclude.

Cricket batters anecdotally report that they make fine adjustments to their wrist orientation in an effort to ensure the ball is directed away from opposition fielders when they hit it. Future studies may be able to uncover whether the ability to ‘park’ gaze at the anticipated location of bat-ball contact provides some form of functional advantage that may result in more efficacious hitting and/or a decreased likelihood that the batter misjudges the precision and accuracy of the interceptive action (Mann *et al*., 2013, p. 10).

I conclude that the challenge from expertise is not, at present, successful. This is true even when we restrict the challenge drastically, to real-time expert control of rapid actions.

## Conclusion

I began this article by noting a truism of folk psychology: human agents routinely exercise conscious control over action. Is the truism true? If the above discussion is on track, we have reason to believe it is. I have elucidated a general model of overt action control that gives a place not only to the broadly implementational aspects of overt action control, but also to executive aspects. Importantly, these extend past the formation of plans and intentions. I have argued that executive processes, including the direction of attention and the filling in and on-line revision of intentions, are important for the on-line control of overt action as well. Further, I have argued that the best way to conceive of conscious control is as a subset of overt action control. In my view, an agent exercises conscious control along one of two dimensions. An agent exercises conscious control along either the executive or implementational dimension if conscious executive or conscious implementational processes make a critical causal contribution to that agent's exercise of overt action control.

This account need not and does not deny the importance of nonconscious processes to overt action control, and indeed, to conscious control. Even so, I have offered evidence favouring scepticism about the import of recent zombie challenges—arguments that purport to show that though it seems (either phenomenally or epistemically) to us that overt action is controlled by conscious states and processes, in fact overt action is controlled by nonconscious (zombie) states and processes. I have argued that the scope of these challenges is more restricted than we might initially think, and that even more restricted zombie challenges are, at present, unsuccessful. Although many details remain unknown, conscious states and processes of various types appear to make critical causal contributions to the control human agents routinely exercise over action.

## Footnotes

1 I speak of the action-production system (singular) merely for convenience.

2 My use of the term motor control, as opposed to overt action control, refers here to the control of bodily movement considered in abstraction from high-level states (like intentions and desires).

3 Importantly, the learning process is throughout influenced by the biomechanical constraints of the individual's speech-production system.

4 One way an intention might do so is by specifying the expected feedback in a certain sensory format. Although it is plausible that motor commands are couched in a motoric format, higher-level intentions do not seem to be restricted in the same way (see Pacherie, 2008).

5 One might argue that this is not an example of intentions structuring feedback, but simply of low-level elements such as efferent copies of motor commands structuring feedback. This kind of objection seems to depend on a view according to which intentions are modular with respect to the visuomotor system, and thus have no direct influence on its functioning. Wayne Wu convincingly argues that this view is false—intentions penetrate vision. I refer readers to his paper (Wu, 2013b).

6 For a recent discussion of control over action that appears broadly sympathetic, see Fridland, 2014. Some earlier studies indicate that autistic agents are impaired on tasks that seem to measure executive capacities, such as the Tower of Hanoi and the Tower of London tasks (Pennington and Ozonoff, 1996). But these results are not replicated in computerised versions of these tasks, suggesting that the earlier failures are due to motor elements essential for non-computerised versions of these tasks. With that said, the claim that autistic agents suffer primarily from implementational deficits remains controversial: see Stoit *et al*., 2013, for discussion. Thanks to an anonymous referee for suggesting I be more explicit about this issue.

7 For further evidence of functional integration between executive and implementational dimensions, see a recent review by Ondobaka and Bekkering (2012): they cover a wide range of evidence indicating that ‘conceptual goals apparently sit atop the perception-guided level in the hierarchical control of overt behaviour’ (2012, p. 3).

8 It is important to note that this way of conceptualising conscious control makes no commitments to the function of consciousness, as that term is normally conceptualised. Those searching for the function of consciousness usually seek to find the proprietary causal contribution consciousness makes (see Morsella *et al*., 2005; Rosenthal, 2008). This search has value, but to my mind it neglects the variety of ways consciousness might contribute: even if consciousness makes no proprietary contribution, various aspects of consciousness might make important contributions in some circumstances. Mapping those contributions is an important task in itself, and might support accounts of the proprietary function(s) of consciousness, if any there be.

9 A further issue concerns the facts that conscious states and events are more than blind cogs in a causal system—they possess phenomenal properties, and according to many, intentional content. Arguably, this is important for assessing the kind of contribution they make, insofar as we want the causal role of some aspect of consciousness to accord with the content or character of that aspect. Consider, for example, a causal and conscious system S which has a part V that is closely associated with (is identical to, is the supervenience base of, or whatever) visual phenomenal properties—properties such as colour, shape, and luminance. Now suppose we discover no correlation between the operation of V and anything that looks like responsiveness to the various visual phenomenal properties associated with it (save reports about visual phenomenology). Instead, we find close correlations between the operations of V and the contractions of S's quadriceps. We might think that even though V makes critical causal contributions to the contractions of S's quadriceps, something is horribly wrong with S.

10 I offer further discussion of relevant issues in Shepherd, 2015.

11 For book-length treatments of the relevant evidence, see Milner and Goodale, 1995, 2004. For recent positive discussions of the dual-visual systems view, see Milner and Goodale, 2008, and Clark, 2009. For a recent critical discussion, see Schenk and McIntosh, 2010.

12 In my view, such a possibility is the tip of an iceberg. In other work I have emphasised the possible importance of non-visual aspects of consciousness for overt action control, such as conscious trying (Shepherd, 2015). There is not space to rehash that discussion here.

13 See Montero, 2010, for a nice discussion of related issues.

14 A nice example of this comes from Perkell *et al*.'s discussion of the motor control of speech. They write, ‘The speech movement control system must combine the influences of robust segmental mechanisms that do not rely on closed-loop auditory feedback with more labile suprasegmental mechanisms that may use relatively simple auditory information closed-loop. That is, the planed acoustic trajectory is influenced by adjustments in suprasegmental parameters that affect intelligibility, such as average sound level, speaking rate, the degree of prosodically based f0 and SPL inflection and clarity of individual sounds. To guide such adjustments, the speaker uses auditory information to assess factors such as the ambient noise level or quality of a phone line (along with other information such as the listener's view of the speaker's face and the listener's knowledge of the language and subject matter). On the basis of this information, the speaker makes relatively rapid adjustments in the average ‘postural’ (baseline) settings of the underlying respiratory, laryngeal and supraglottal mechanisms’ (2000, p. 239). Not all of these parameter adjustments will be or need be influenced by conscious processes, but it is plausible that some could be.

15 Interestingly, expert prediction is not simply the result of visual processing. Predictive abilities improve as motor involvement in the generation of the prediction increases (Mann *et al*., 2010).

16 An anonymous referee makes the (to my mind, good) point that even if evidence indicates that the phenomenology of batting depends in part on reconstructive processes, the inference that such phenomenology is systematically mistaken is problematic. For since much visual phenomenology looks to result from the interaction of predictive and reconstructive processes with bottom-up sensory processes, such an inference creates pressure to accept that most visual phenomenology is systematically mistaken. Quoting the referee: ‘I am not sure this bullet is worth biting.’
